# Comprehensive Cardiac Magnetic Resonance to Detect Subacute Myocarditis

**DOI:** 10.3390/jcm11175113

**Published:** 2022-08-30

**Authors:** Jan M. Brendel, Karin Klingel, Jens Kübler, Karin A. L. Müller, Florian Hagen, Meinrad Gawaz, Konstantin Nikolaou, Simon Greulich, Patrick Krumm

**Affiliations:** 1 Department of Radiology, Diagnostic and Interventional Radiology, University of Tübingen, Hoppe-Seyler-Straße 3, 72076 Tübingen, Germany; 2 Cardiopathology, Institute for Pathology and Neuropathology, University of Tübingen, Liebermeisterstraße 8, 72076 Tübingen, Germany; 3 Department of Internal Medicine III, Cardiology and Angiology, University of Tübingen, Otfried-Müller-Straße 10, 72076 Tübingen, Germany

**Keywords:** acute myocarditis, subacute myocarditis, magnetic resonance imaging, CMR, LGE, T_1_ mapping, T_2_ mapping, ECV, Lake Louise criteria

## Abstract

**(1) Background:** Compared to acute myocarditis in the initial phase, detection of subacute myocarditis with cardiac magnetic resonance (CMR) parameters can be challenging due to a lower degree of myocardial inflammation compared to the acute phase. **(2) Objectives:** To systematically evaluate non-invasive CMR imaging parameters in acute and subacute myocarditis. **(3) Methods:** 48 patients (age 37 (IQR 28–55) years; 52% female) with clinically suspected myocarditis were consecutively included. Patients with onset of symptoms ≤2 weeks prior to 1.5T CMR were assigned to the acute group (*n* = 25, 52%), patients with symptom duration >2 to 6 weeks were assigned to the subacute group (*n* = 23, 48%). CMR protocol comprised morphology, function, 3D-strain, late gadolinium enhancement (LGE) imaging and mapping (T_1_, ECV, T_2_). **(4) Results:** Highest diagnostic performance in the detection of subacute myocarditis was achieved by ECV evaluation either as single parameter or in combination with T_1_ mapping (applying a segmental or global increase of native T_1_ > 1015 ms and ECV > 28%), sensitivity 96% and accuracy 91%. Compared to subacute myocarditis, acute myocarditis demonstrated higher prevalence and extent of LGE (AUC 0.76) and increased T_2_ (AUC 0.66). **(5) Conclusions:** A comprehensive CMR approach allows reliable diagnosis of clinically suspected subacute myocarditis. Thereby, ECV alone or in combination with native T_1_ mapping indicated the best performance for diagnosing subacute myocarditis. Acute vs. subacute myocarditis is difficult to discriminate by CMR alone, due to chronological connection and overlap of pathologic findings.

## 1. Introduction

The diagnosis of subacute myocarditis remains challenging due to various reasons. In contrast to acute myocarditis, patients with subacute myocarditis may present with rather mild or non-specific symptoms. Primary clinical workup may reveal non-pathologic ECG, preserved or just slightly impaired left-ventricular function, as well as more discrete laboratory findings than in the acute phase of inflammation [1,2].

In clinical practice, CMR has emerged as a diagnostic tool to confirm clinically suspected myocarditis due to its ability of non-invasive multiparametric tissue characterization [2,3,4,5,6,7]. However, as myocardial edema decreases and CMR lesions become more diffuse, the decrease of myocardial inflammation during the transition from the acute to the subacute phase still poses a challenge for diagnosing subacute myocarditis [8,9,10,11,12].

Yet, the correct diagnosis is of high importance at this stage of myocarditis and might be crucial regarding patients’ recovery. Patients may both need general supportive therapy, heart failure medication and abstinence from competitive sports in order not to risk a transition to chronic myocarditis or even dilated cardiomyopathy (DCM), considerably worsening the course [13,14].

The purpose of this study was to systematically evaluate non-invasive CMR parameters for detection of subacute myocarditis and to test their diagnostic performance compared to acute myocarditis and healthy controls.

## 2. Materials and Methods

### 2.1. Study Population

In this single center study, 332 consecutive patients with clinically suspected myocarditis underwent CMR imaging from October 2019 to May 2022 and were prospectively evaluated. N = 265 patients were excluded due to a symptom duration >6 weeks or other final CMR diagnosis of non-ischemic cardiomyopathy. N = 14 patients were excluded due to a history of coronary artery disease, angiographic evidence of coronary artery disease (CAD) or pre-existing valve disease. N = 5 patients were excluded due to incomplete dataset acquisition. Datasets of *n* = 48 myocarditis patients (age 37 (IQR 28–55) years; 52% female) were finally evaluable.

Based on symptom onset prior to CMR, patients were assigned to two groups: (1) acute group ≤2 weeks (*n* = 25, 52%), (2) subacute group >2 to 6 weeks (*n* = 23, 48%). Inclusion criteria were as adapted from the ESC Task Force Criteria for clinically suspected myocarditis [5]: (1) novel onset or worsening of heart failure symptoms or symptoms indicative of myocarditis (dyspnea, drop in performance, fever, chest pain, palpitations); (2) medical history with recent viral infection; (3) pathological results in basic diagnostics suggestive of myocardial damage (elevated troponin and/or NT-proBNP, abnormal electrocardiogram, echocardiographic impaired LV function).

Additional endomyocardial biopsy for reference standard diagnosis confirmation was performed according to current clinical indications [15] in 6 patients of the acute group and 2 patients of the subacute group within median 1 day of CMR.

Patients’ symptoms, cardiovascular risk profiles and laboratory values were recorded; 15 healthy volunteers served as a control group (Supplementary File S1). All subjects gave written informed consent, and the Institutional Review Board approved the study protocol.

### 2.2. CMR Image Aqcuisition

CMR examinations were performed on a 1.5T scanner (MAGNETOM Aera, SIEMENS Healthcare, Erlangen, Germany). CMR protocol comprised late gadolinium enhancement (LGE) imaging and mapping (T_1_, ECV, T_2_) as well as additional detection of pericardial effusion and acquisition of morphology, volumetry, and strain. For functional assessment, steady state free precession (SSFP) CINE loops in vertical and horizontal long-axis as well as short-axis orientation were performed. T_1_ mapping was performed native and 15–20 min post contrast agent administration with a MOLLI sequence 5(3)3 with generation of 3 short axis T_1_ maps (apical, mid, basal). T_2_ mapping was performed before contrast media application with a T_2_ prepared SSFP sequence in 3 short axis slices (apical, mid, basal) with 2D inversion recovery (IR) gradient recovery echo (GRE) sequence for late enhancement imaging 10 min after intravenous administration of 0.15 mmol Gadobutrol (Gadovist, Bayer Healthcare, Leverkusen, Germany) per kg body weight.

Detailed sequence parameters for CMR imaging are given in detail in Supplementary File S1.

### 2.3. CMR Image Analysis

Image analysis was performed by two experienced CMR readers in consensus using dedicated software (cvi42 Version 5.13, CVI Circle Cardiovascular Imaging, Calgary, AB, Canada) and according to the Society for Cardiovascular Magnetic Resonance (SCMR) recommendations [16,17]. *Functional assessment* was performed in a stack of SAX slices with semi-automated contouring of endocardial and epicardial borders, with manual re-adjustment if necessary; cutoff values according to [18]. *3D-Strain analysis* for global radial (GRS), circumferential (GCS) and longitudinal strain (GLS) was performed using post-processing CMR feature tracking of 4CV and SAX cine loops; cutoff values according to [19]. *LGE imaging* was evaluated qualitatively and semi-quantitatively: LGE patterns (linear vs. patchy) were qualitatively assessed and localized (septal mid-myocardial vs. subepicardial) and assigned to the myocardial segments according to the 17 segment-model of the American Heart Association [20]. Semi-quantitative evaluation of LGE fraction of left-ventricular (LV) myocardial mass was performed with a threshold of ≥2 standard deviations (SD) above remote myocardium [16].

*T_1_ and T_2_ mapping* was evaluated in a segmental and global approach; values were considered elevated if above 2 standard deviations of a healthy in-house control group performed at the same 1.5T scanners (T_1_ > 1053; T_2_ > 51 ms). For descriptive statistics, ECV values above 30% were considered as definitely increased [21,22,23]. For diagnostic evaluation, criterions derived from receiver-operating (ROC) curves were used as cutoff values. A combination of 2018 expert recommendations for updated CMR criteria in acute and subacute myocardial inflammation (‘Lake Louise criteria’; 2018 LLC) were evaluated in each case: (1) myocardial edema (elevated T_2_) and (2) non-ischemic injury (elevated T_1_, and/or ECV, and/or LGE) [4].

### 2.4. Endomyocardial Biopsy Protocol

Endomyocardial biopsies were performed in selected patients according to current ESC diagnostic guidelines [5]. At least five right-ventricular biopsies were taken followed by a comprehensive cardiopathological workup. For details see Supplementary File S1.

### 2.5. Statistical Analysis

Normality of data was tested using the Kolmogorov–Smirnov test. Continuous, nonparametric variables are indicated as median (interquartile range). Categorical data are indicated as frequency (percentage %). For unpaired group comparison Mann–Whitney *U* test was performed in continuous nonparametric data; Fisher’s exact test was performed in categorical data (JMP, Version 16, SAS Institute Inc., Heidelberg, Germany). Receiver operating characteristic (ROC) curves were generated for comparison of LGE and mapping parameters in patients with acute and subacute myocarditis as well as in controls using the method of Delong et al. [24] (MedCalc, Version 18, MedCalc Software Ltd., Ostend, Belgium). Global level of significance *α* was set to 5%. Local level of significance (*α*_loc_) for each test with dependent variables was corrected according to the Bonferroni equation according to *k = 85* performed comparisons: α _loc_ = α _glob_/*k* = 0.0006.

## 3. Results

### 3.1. Patient Characteristics

Table 1 depicts the study’s patient characteristics. All patients were symptomatic (at least 1 symptom) at the time of diagnostic work-up: 20 (42%) demonstrated dyspnea, 17 (35%) chest pain, 16 (33%) fever, 16 (33%) fatigue, 12 (25%) angina pectoris and 2 (4%) peripheral edema. About one quarter of all patients (*n* = 11, 23%) were ≥NYHA III. One female patient of the acute group had COVID-19.

Nearly half of all patients (*n* = 22, 46%) had impaired LV-EF: 11 (44%) patients with acute myocarditis; 11 (48%) patients with subacute myocarditis, Table 2. EMB confirmed clinically suspected myocarditis in 8 cases and revealed the presence of parvovirus B19 and human herpesvirus 6 in 2 cases of the acute group and in 1 case of the subacute group, as well as Epstein–Barr virus in 1 case of each group. One case of the subacute group showed all three virus types. Electrocardiogram (ECG) revealed ST-segment elevation in 6 (24%) and T-wave inversion in 7 (28%) acute myocarditis patients vs. none of the subacute myocarditis patients, *p* = 0.012 and *p* = 0.007, respectively. Troponin I revealed a median of 508 ng/L (IQR 114–4391) in acute vs. 39 ng/L (IQR 17–118) in subacute myocarditis patients, *p* < 0.0001. Troponin >5 times beyond the reference range was found in 14 (56%) patients of the acute group vs. in 1 (4%) patient of the subacute group, *p* < 0.0001. Likewise, NT-proBNP was increased in 20 (80%) patients of the acute group vs. in 7 (30%) patients of the subacute group, *p* < 0.001. C-reactive protein (CRP) was higher in the acute than subacute group, 5 mg/dL (IQR 0.5–8) vs. 0.3 mg/dL (IQR 0.1–1), *p* = 0.001.

### 3.2. Subacute Myocarditis vs. Controls

**Figure 1:**
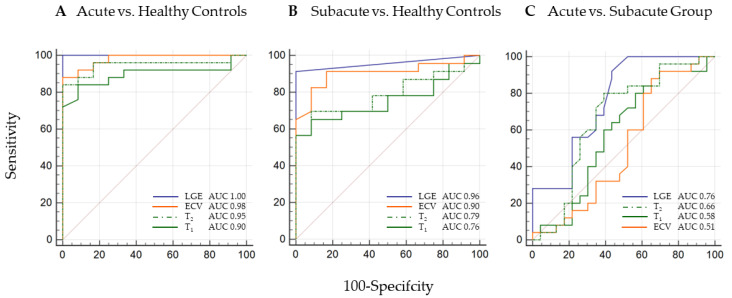
**CMR Parameter ROC Curves for Discrimination of Subacute Myocarditis from Healthy Controls and Acute Myocarditis.** (**A**) ROC curves demonstrate excellent areas under the curve (AUCs) for all four tissue characterization parameters for discrimination of acute myocarditis from healthy controls. (**B**) In the discrimination of subacute myocarditis from healthy controls, LGE and ECV performed best with AUCs of 0.96 (*p* < 0.0001) and 0.90 (*p* < 0.0001) respectively; 0.79 (*p* < 0.001) for T_2_ with a criterion of >49 ms; 0.76 (*p* = 0.002) for T_1_ with a criterion of >1015 ms. (**C**) For comparison of acute from subacute myocarditis, the areas under the curve (AUCs) were 0.76 (*p* < 0.001) for LGE with a criterion of >2, 8% of LV myocardial mass; 0.66 (*p* = 0.049) for T_2_ with a criterion of >51 ms; T_1_ and ECV showed no significant differences. The diagonal line course indicates difficult discrimination of acute vs. subacute myocarditis by CMR alone.

For discrimination of subacute myocarditis from healthy controls, LGE imaging and ECV mapping demonstrated the highest AUCs with 0.96 (*p* < 0.0001) for LGE and 0.90 (*p* < 0.0001) for ECV; T_2_ and T_1_ mapping performed slightly inferior 0.79 (*p* < 0.001) for T_2_ and 0.76 (*p* = 0.002) for T_1_. AUCs revealed a criterion of >1015 ms for T_1_ and of >49 ms for T_2_, Figure 1.

**Figure 2:**
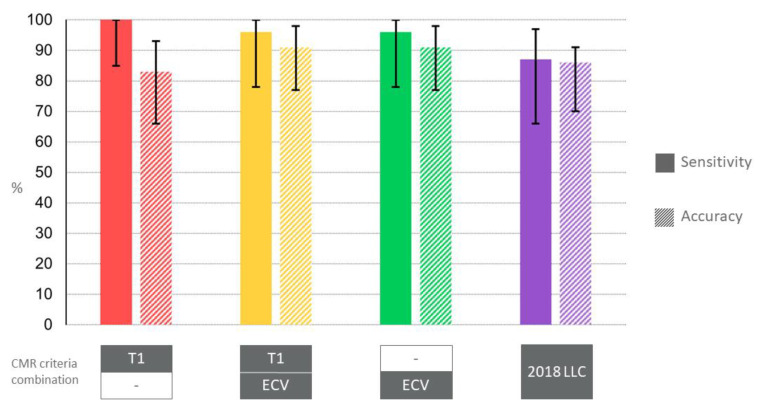
**Diagnostic Performance of CMR Criteria Combination for Discrimination of Subacute Myocarditis from Healthy Controls.** The best diagnostic performance in the detection of subacute myocarditis and the discrimination from healthy controls was achieved by both ECV evaluation alone or in combination with T_1_ mapping, demonstrating a sensitivity of 96% (CI 78–100) and an accuracy of 91% (CI 77–98). A segmental or global increase of native T_1_ > 1015 ms and ECV > 28% was applied, derived from ROC analysis. LLC = Lake Louise criteria.

The best diagnostic performance in the detection of subacute myocarditis and the discrimination from healthy controls was achieved by both ECV evaluation alone or in combination with T_1_ mapping (applying a segmental or global increase of native T_1_ > 1015 ms and ECV > 28%), demonstrating a sensitivity of 96% (CI 78–100) and an accuracy of 91% (CI 77–98), see Figure 2.

T_1_ mapping had a sensitivity of 100% (CI 85–100) with lowest specificity of 50% (CI 21–79). T_2_ mapping showed a sensitivity of 87% (CI 66–97) and an accuracy of 83% (66–93); 2018 expert recommendations (LLC) resulted in a sensitivity of 87% (CI 66–97) and an accuracy of 86% (CI 70–95). LGE had the lowest sensitivity of 61% (CI 39–80), but the highest specificity with 100%. Diagnostic performances of CMR parameters are depicted in Table 3 and Figure 2.

### 3.3. Acute Myocarditis and Subacute Myocarditis

**Figure 3:**
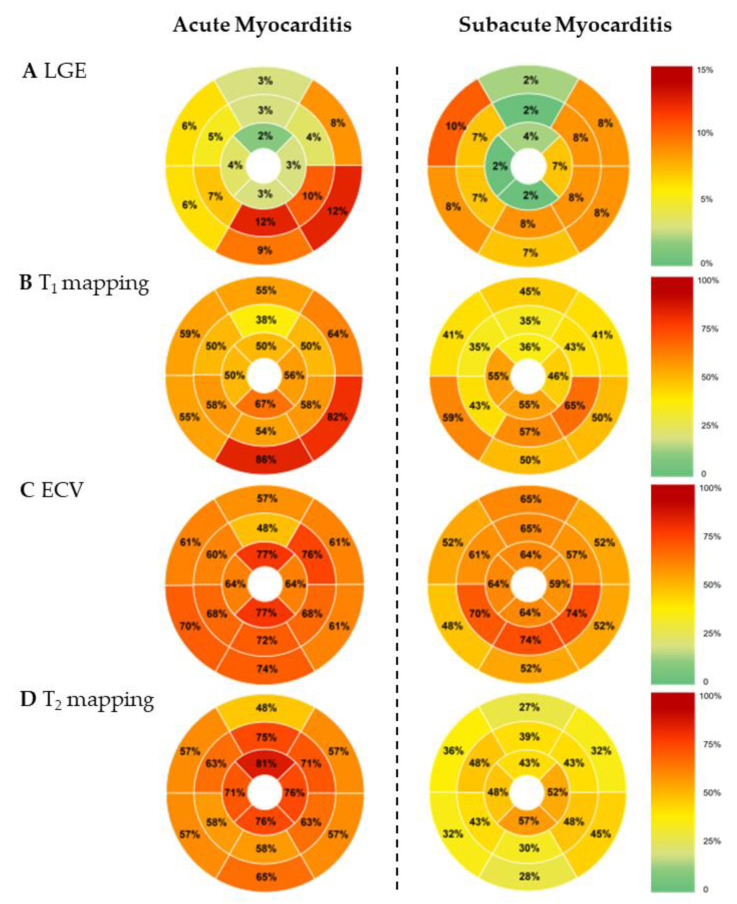
Location of LGE and Elevated Mapping Parameters per AHA Segments. Heatmapped 17-segment-model schemes (according to the American Heart Association) illustrate the percentage frequency of the occurrence of (**A**) LGE, (**B**) elevated T_1_, (**C**) elevated extracellular volume fraction (ECV) and (**D**) elevated T_2_.

CMR findings are summarized in Table 2 and Table 4. Pericardial effusion >5 mm was present in 12 (48%) patients with acute myocarditis vs. in 4 (17%) patients of the subacute group, *p* = 0.022. LGE was present in 22 (88%) of acute myocarditis patients vs. in 14 (61%) of the subacute group, *p* = 0.028. LGE extent was 5% (IQR 3–9) of LV myocardial mass in acute vs. 3% (IQR 0–5) in subacute myocarditis, *p* = 0.002. Linear subepicardial LGE pattern was the most common pattern in both groups. Global T_2_ was increased in 20 (80%) patients with acute myocarditis vs. in 10 (43%) patients of the subacute group, *p* = 0.008. Acute myocarditis patients had median 10 (IQR 8–15) T_2_ elevated segments vs. 6 (IQR 2–11) in subacute patients, *p* = 0.048. Segmental distribution of LGE and elevated mapping parameters are illustrated in Figure 3.

### 3.4. Acute Myocarditis vs. Subacute Myocarditis

**Figure 4:**
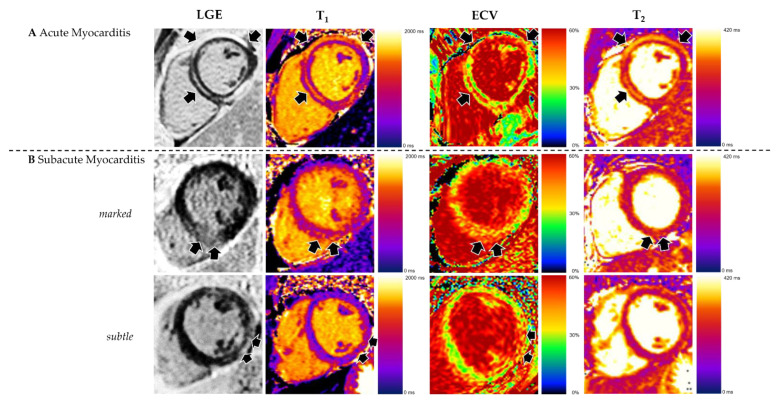
**Appearance of Acute and Subacute Myocarditis in CMR.** (**A**) Acute myocarditis often demonstrates more obvious alterations of tissue characterization parameters including high prevalence and extent of late gadolinium enhancement (LGE) and elevated T_2_. (**B**) Subacute myocarditis can manifest with marked occurrence of LGE and elevated T_1_, ECV and T_2_; but in many cases may demonstrate rather subtle changes in tissue characterization.

For discrimination of acute from subacute myocarditis, the areas under the curve (AUCs) were 0.76 (*p* < 0.001) for LGE with a criterion of >2.8% of LV myocardial mass; 0.66 (*p* = 0.049) for T_2_ with a criterion of >51 ms. T_1_ and ECV did not differ significantly, as shown in Figure 1. Typical CMR examples of acute and subacute myocarditis are illustrated in Figure 4.

## 4. Discussion

This prospective study systematically evaluated non-invasive CMR imaging parameters for the detection of subacute myocarditis compared to acute myocarditis and healthy controls. Clinically suspected subacute myocarditis could be sensitively detected by (1) ECV evaluation alone or (2) in combination with T_1_ mapping or (3) T_2_ mapping.

### 4.1. Subacute Myocarditis vs. Controls

***ECV and native T_1_ mapping*.** Regarding the detection of subacute myocarditis, both ECV alone or in combination with T_1_ mapping yielded the best sensitivity (96%) and accuracy (91%). Radunski et al. reported the best diagnostic accuracy for global myocardial ECV >27% in diagnosis of acute myocarditis [25]; Luetkens et al. applied an ECV cutoff of 28.8% achieving an accuracy of 74% in diagnosing myocarditis [26]. This is in line with the ECV cutoff of 28% for subacute myocarditis which we found in our study. ECV has shown to be capable of detecting subtle myocardial alternations including fibrosis and therefore is especially beneficial when LGE is not attainable [25,27]. Furthermore, ECV has proven to be an independent CMR parameter robustly associated with outcome in myocardial fibrosis [28], and to be the best imaging biomarker of acute myocarditis burden also in dual-energy computed tomography (DECT), allowing an early prediction of the occurrence of cardiac complications [29,30].

Segmental or global elevation of T_1_ values above the cutoff of 1015 ms showed an excellent sensitivity of 100%, however, with only low specificity of 50% (Table 3). Specificity improved to 83%, additionally including ECV. Moreover, the combination of T_1_ mapping with ECV has shown high sensitivity (96%) and accuracy (91%) with an additional benefit of diversification compared to ECV evaluation as a single parameter. T_1_ elevation above 990 ms has been previously proposed for detection of inflammation in acute myocarditis with good diagnostic performance [31,32]. In our study, this cutoff would have resulted in a substantial rise of the false positive rate and a decrease of the positive predictive value.

***T_2_ mapping*.** Evaluation of T_2_ showed lower diagnostic performance in single parameter analysis compared to T_1_ and ECV with a sensitivity of 87% and accuracy of 83%, applying a segmental or global increase of >49 ms for T_2_. Performing ECV in addition to T_2_ improved the specificity. This result supports the approach that T_1_-based ECV and T_2_ mapping have complementary diagnostic value as reflected by current expert recommendations for the diagnostic management of patients with suspected myocarditis [33,34].

***2018 Expert Recommendations (Lake Louise criteria).*** Application of 2018 LLC resulted in a sensitivity of 87% and an accuracy of 86% and thus provided less accurate diagnosis compared to the duo of ECV + T_1_ mapping. However, 2018 LLC showed decent diagnostic performance, considering being designed for detection of myocarditis in the acute and subacute phase [35].

***Late Gadolinium Enhancement.*** LGE had the lowest single parameter detection rate with a sensitivity of 61% and an accuracy of 74%. Lagan et al. reported similar compiled sensitivity and accuracy for LGE in myocarditis of 63 and 72%, respectively [7]. A meta-analysis by Kotanidis et al. also stated comparable numbers with a sensitivity of 68% [36]. Considering the presented superior sensitivity of mapping techniques, the question arises whether implication of LGE is of additional benefit and whether LGE may also be omitted in a comprehensive CMR protocol in suspected subacute myocarditis. Hereby, two important upsides of LGE are of note: First, LGE with its characteristic non-ischemic patterns showed the highest specificity in this study in line with previous studies [36]. Second, occurrence of LGE has also shown to be of predictive value for major adverse cardiac events and outcome [37]. Hereby, especially septal LGE is associated with worsening of the course compared to subepicardial lateral LGE [38,39,40,41].

### 4.2. Acute vs. Subacute Myocarditis

Pathological ECG findings and blood results were less conspicuous and less common in patients with subacute myocarditis. Pericardial effusion, elevated T_2_ values as well as LGE prevalence were more frequent and more pronounced in the acute myocarditis group. The AUCs of LGE and T_2_ showed the best discrimination of both groups with an LGE extent > 2.8% of LV myocardial mass and T_2_ > 51 ms. This might be explained by a decrease of inflammation from the acute to the subacute phase [42]. T_1_ mapping has proven to be a sensitive marker for myocardial disease of different entities [33,43], but seems limited for discrimination of acute from chronic processes [44]. ROC analysis indicated difficult discrimination of acute vs. subacute myocarditis by CMR alone due to an overlap of the pathologic findings.

## 5. Limitations

As a limitation of this study, endomyocardial biopsy (EMB) was not performed in all patients, but only for diagnosis confirmation in eight ambiguous cases after a careful risk-benefit analysis according to current indications [15]. The overall sample size is limited. T_1_ and T_2_ mapping values are applicable for the specific scanner and sequence type used in this study and not generalizable over all vendors, scanners, and sequence types.

## 6. Clinical Implications

Many patients with inflammatory cardiomyopathy tend to present with a latency of several weeks since symptom onset. According to 2013 ESC recommendations, EMB is recommended for definite diagnosis of myocarditis [5]. However, in clinical routine, EMB is not always performed due to various limitations (e.g., availability, invasiveness, sampling error) and CMR has emerged as a tool to non-invasively characterize myocardial tissue [3,4,6]. Therefore, the 2013 ESC recommendations are expected to be revised in the next years [5,12].

This study provides CMR data on detection of clinically suspected subacute myocarditis. In view of the fact that CMR has recently been accepted as a workflow for non-invasive confirmation of clinically suspected myocarditis [3,14], this may help to detect ongoing myocardial inflammation.

In contrast, unrecognized inflammation in CMR may falsely underestimate subacute myocarditis and lead to incorrect declaration of myocarditis as cured. As a consequence, subacute myocarditis may progress to chronic myocarditis or dilated cardiomyopathy instead of healing to complete restitutio ad integrum.

## 7. Conclusions

A comprehensive CMR approach allows reliable diagnosis of clinically suspected subacute myocarditis. Thereby, ECV alone or in combination with native T_1_ mapping indicated the best performance for diagnosing subacute myocarditis.

Acute vs. subacute myocarditis is difficult to discriminate by CMR alone, due to chronological connection and overlap of pathologic findings.

## Figures and Tables

**Table 1:** Patient characteristics.

Characteristic	Acute Group *n* = 25 (52)	Subacute Group *n* = 23 (48)	*p*-Value
Age [yrs]	32 (22–45)	48 (30–63)	
Female	12 (48)	13 (56)	
BMI [kg/m²]	25 (23–29)	24 (21–28)	
Duration of symptoms [days]	3 (2–6)	29 (21–32)	
Symptoms			
Dyspnea	12 (48)	8 (34)	n.s.
Chest pain	12 (48)	5 (22)	n.s.
Fever	8 (32)	8 (34)	n.s.
Fatigue	7 (28)	9 (39)	n.s.
Angina pectoris	6 (24)	6 (26)	n.s.
Peripheral edema	1 (4)	1 (4)	n.s.
NYHA-Classification			
NYHA I	13 (52)	15 (65)	n.s.
NYHA II	4 (16)	5 (22)	n.s.
NYHA III	4 (16)	2 (9)	n.s.
NYHA IV	4 (16)	1 (4)	n.s.
CVRF			
Arterial Hypertension	3 (12)	7 (30)	n.s.
Diabetes	2 (8)	3 (13)	n.s.
Dyslipidemia	2 (8)	3 (13)	n.s.
Smoking	2 (8)	2 (9)	n.s.
Obesity	5 (20)	4 (17)	n.s.
ECG findings			
Tachycardic sinus rhythm	1 (4)	1 (4)	n.s.
Left bundle branch block	1 (4)	0	n.s.
AV node block type III	0	1 (4)	n.s.
ST-segment elevation	6 (24)	0	0.012
T-wave inversion	7 (28)	0	0.007
Blood results			
Troponin [ng/L]	508 (114–4391)	39 (17–118)	<0.0001
Troponin elevated ♂ > 57 ♀ > 37 [ng/L]	22 (88)	13 (56)	0.013
Troponin elevated >3 times	19 (76)	5 (22)	<0.001
Troponin elevated >5 times	14 (56)	1 (4)	<0.0001
NT-proBNP [ng/L]	650 (175–1108)	127 (78–455)	<0.0001
NT-proBNP elevated >300 [ng/L]	20 (80)	7 (30)	<0.001
CRP [mg/dL]	5 (0.5–8)	0.3 (0.1–1)	0.001
CRP elevated >0.5 [mg/dL]	18 (72)	7 (30)	0.004
Leucocytes [1/µL]	11,300 (9100–14,300)	8600 (7900–10,000)	0.011
Leucocytes elevated >10,300 [1/µL]	13 (52)	4 (17)	0.012
EMB, performed in *n* = 8 (100) patients	*n* = 6 (75)	*n* = 2 (25)	
Presence of viral genomes (multiple possible)			
Parvovirus B19	2 (33)	1 (50) *	n.s.
Human herpesvirus 6	2 (33)	1 (50) *	n.s.
Epstein-Barr virus	1 (16)	1 (50) *	n.s.

Values are given as frequency (percentage %) or median (interquartile range); *p*-values ≤ 0.05 were considered as significant; n.s. = not significant; BMI = body mass index; NYHA = New York Heart Association; CVRF = cardiovascular risk factors; ECG = electrocardiogram; AV = atrioventricular; NT-proBNP = N-terminal pro-B-type natriuretic peptide; CRP = C-reactive protein; EMB = endomyocardial biopsy; * One case of the subacute group showed all three virus types.

**Table 2:** CMR results in acute and subacute myocarditis.

Parameter	Acute Group *n* = 25 (52)	Subacute Group *n* = 23 (48)	*p*-Value
Morphology [mm]			
LV-EDD 4-chamber view	50 (46–56)	50 (47–54)	n.s.
RV-EDD 4-chamber view	42 (40–48)	44 (40–47)	n.s.
IVS	8 (7–10)	8 (7–10)	n.s.
Pericardial effusion			
Pericardial effusion [mm]	5 (2–6)	3 (2–4)	n.s.
Pericardial effusion >5 mm	12 (48)	4 (17)	0.022
Volumetry (LV)			
EF [%]	58 (45–63)	59 (47–64)	n.s.
EF reduced ♂ > 57 ♀ < 58	11 (44)	11 (48)	n.s.
SV [mL]	81 (60–101)	90 (78–108)	n.s.
Indexed SV [mL/m²]	42 (32–48)	51 (45–60)	0.009
Indexed SV reduced ♂ > 43 ♀ < 40	12 (48)	4 (17)	0.022
EDV [mL]	155 (125–190)	167 (132–192)	n.s.
Indexed EDV [mL/m²]	73 (68–96)	92 (79–103)	0.034
Indexed EDV elevated ♂ > 100 ♀ > 95	7 (28)	9 (39)	n.s.
ESV [mL]	61 (44–97)	77 (54–100)	n.s.
Indexed ESV [mL/m²]	32 (25–52)	42 (32–49)	n.s.
Indexed ESV elevated ♂ > 39 ♀ > 35	9 (36)	12 (52)	n.s.
Peak strain (%)			
Global Radial strain	27 (16–32)	29 (23–34)	n.s.
Global Radial strain reduced <22	9 (36)	4 (17)	n.s.
Global Circumferential strain	−18 (−20 to −15)	−18 (−21 to −16)	n.s.
Global Circumferential strain reduced >−13	6 (24)	4 (17)	n.s.
Global Longitudinal strain	−12 (−15 to −10)	−13 (−15 to −12)	n.s.
Global Longitudinal strain reduced >−9	5 (20)	0	0.008

Values are given as frequency (percentage %) or median (interquartile range); *p*-values ≤ 0.05 were considered as significant; n.s. = not significant; indexed data are normalized to body surface area; LV = left-ventricular; RV = right-ventricular; EDD = end-diastolic diameter; IVS = interventricular septum; EF = ejection fraction; SV = stroke volume; EDV = end-diastolic volume; ESV = end-systolic volume.

**Table 3:** Diagnostic Performance of CMR Criteria Combinations for Confirmation of Clinically Suspected Diagnosis of Subacute Myocarditis.

Parameter(s)	Sensitivity	Specificity	Positive Predictive Value	Negative Predictive Value	Accuracy
Single parameter					
T_1_ relaxation times	100	50	79	100	83
ECV	96	83	92	91	91
T_2_ relaxation times	87	75	87	75	83
LGE	61	100	100	57	74
Combined parameters					
T_1_ + ECV	96	83	92	91	91
Lake Louise criteria	87	83	91	77	86
ECV + T_2_	83	92	95	73	86

Data are percentages. Cutoff values were 1015 ms for T_1_, 28% for ECV, 49 ms for T_2_ and 0% for LGE. Global or segmental elevation over the cutoff value was considered positive for subacute myocarditis. In parameter combination, only elevation in every parameter resulted in a positive count.

**Table 4:** CMR Tissue Characterization of Acute and Subacute Myocarditis.

Parameter(s)	Acute Group *n* = 25 (52)	Subacute Group *n* = 23 (48)	*p*-Value
Late Gadolinium Enhancement (LGE)			
Prevalence	22 (88)	14 (61)	0.028
Number of positive segments	4 (2–5)	2 (0–4)	n.s.
>2 SD [% of LV myocardial mass]	5 (3–9)	3 (0–5)	0.002
Pattern type			
Linear septal mid-myocardial	6 (24)	6 (26)	n.s.
Linear subepicardial	14 (56)	8 (35)	n.s.
Patchy	6 (24)	3 (13)	n.s.
Mapping			
T_1_ global relaxation time [ms]	1069 (1024–1127)	1033 (995–1135)	n.s.
T_1_ global elevated (>1053 ms) *	14 (56)	9 (39)	n.s.
T_1_ elevated in ≥1 segment	22 (88)	21 (91)	n.s.
T_1_ total of elevated segments	9 (5–15)	6 (2–13)	n.s.
ECV global [%]	33 (31–35)	33 (30–36)	n.s.
ECV global elevated (>30%)	22 (88)	15 (65)	n.s.
ECV elevated in ≥1 segment	24 (96)	21 (91)	n.s.
ECV total of elevated segments	10 (7–14)	10 (6–14)	n.s.
T_2_ global relaxation time [ms]	53 (52–56)	51 (48–54)	n.s.
T_2_ global elevated (>51 ms) *	20 (80)	10 (43)	0.008
T_2_ elevated in ≥1 segment	23 (92)	20 (87)	n.s.
T_2_ total of elevated segments	10 (8–15)	6 (2–11)	0.048

Values are given as frequency (percentage %) or median (interquartile range); *p*-values ≤ 0.05 were considered as significant; n.s. = not significant; LGE = late gadolinium enhancement; LV = left-ventricular; ECV = extracellular volume fraction; * >2 SD of control group.

## Data Availability

The datasets analyzed in our study are available from the corresponding author on reasonable request.

## Supplementary Materials

The following supporting information can be downloaded at: https://www.mdpi.com/article/10.3390/jcm11175113/s1, File S1. Notice additional reference [5,45].

## References

[1] Imazio M., Trinchero R. (2008). Myopericarditis: Etiology, management, and prognosis. Int. J. Cardiol..

[2] Lampejo T., Durkin S.M., Bhatt N., Guttmann O. (2021). Acute myocarditis: Aetiology, diagnosis and management. Clin. Med..

[3] Law Y.M., Lal A.K., Chen S., Čiháková D., Cooper L.T., Deshpande S., Godown J., Grosse-Wortmann L., Robinson J.D., Towbin J.A. (2021). Diagnosis and Management of Myocarditis in Children. Circulation.

[4] Ferreira V.M., Schulz-Menger J., Holmvang G., Kramer C.M., Carbone I., Sechtem U., Kindermann I., Gutberlet M., Cooper L.T., Liu P. (2018). Cardiovascular Magnetic Resonance in Nonischemic Myocardial Inflammation: Expert Recommendations. J. Am. Coll. Cardiol..

[5] Caforio A.L.P., Pankuweit S., Arbustini E., Basso C., Gimeno-Blanes J., Felix S.B., Fu M., Heliö T., Heymans S., Jahns R. (2013). Current state of knowledge on aetiology, diagnosis, management, and therapy of myocarditis: A position statement of the European Society of Cardiology Working Group on Myocardial and Pericardial Diseases. Eur. Heart J..

[6] McDonagh T.A., Metra M., Adamo M., Gardner R.S., Baumbach A., Böhm M., Burri H., Butler J., Čelutkienė J., Chioncel O. (2021). 2021 ESC Guidelines for the diagnosis and treatment of acute and chronic heart failure. Eur. Heart J..

[7] Lagan J., Schmitt M., Miller C.A. (2017). Clinical applications of multi-parametric CMR in myocarditis and systemic inflammatory diseases. Int. J. Cardiovasc. Imaging.

[8] Lurz P., Luecke C., Eitel I., Föhrenbach F., Frank C., Grothoff M., de Waha S., Rommel K.P., Lurz J.A., Klingel K. (2016). Comprehensive Cardiac Magnetic Resonance Imaging in Patients with Suspected Myocarditis the MyoRacer-Trial. J. Am. Coll. Cardiol..

[9] Nagel E., Kwong R.Y., Chandrashekhar Y.S. (2020). CMR in Nonischemic Myocardial Inflammation: Solving the Problem of Diagnosing Myocarditis or Still Diagnostic Ambiguity?. J. Am. Coll. Cardiol..

[10] Wheen P., Armstrong R., Daly C.A. (2020). Recent Advances in T_1_ and T_2_ Mapping in the Assessment of Fulminant Myocarditis by Cardiac Magnetic Resonance. Curr. Cardiol. Rep..

[11] Luetkens J.A., Homsi R., Dabir D., Kuetting D.L., Marx C., Doerner J., Schlesinger-Irsch U., Andrié R., Sprinkart A.M., Schmeel F.C. (2016). Comprehensive Cardiac Magnetic Resonance for Short-Term Follow-Up in Acute Myocarditis. J. Am. Hearth Assoc..

[12] Ammirati E., Frigerio M., Adler E.D., Basso C., Birnie D.H., Brambatti M., Friedrich M.G., Klingel K., Lehtonen J., Moslehi J.J. (2020). Management of Acute Myocarditis and Chronic Inflammatory Cardiomyopathy: An Expert Consensus Document. Circ. Heart Fail..

[13] Sagar S., Liu P.P., Cooper L.T. (2012). Myocarditis. Lancet.

[14] Eichhorn C., Greulich S., Bucciarelli-Ducci C., Sznitman R., Kwong R.Y., Gräni C. (2022). Multiparametric Cardiovascular Magnetic Resonance Approach in Diagnosing, Monitoring, and Prognostication of Myocarditis. JACC Cardiovasc. Imaging.

[15] Cooper L.T., Baughman K.L., Feldman A.M., Frustaci A., Jessup M., Kuhl U., Levine G.N., Narula J., Starling R.C., Towbin J. (2007). The Role of Endomyocardial Biopsy in the Management of Cardiovascular Disease. J. Am. Coll. Cardiol..

[16] Schulz-Menger J., Bluemke D.A., Bremerich J., Flamm S.D., Fogel M.A., Friedrich M.G., Kim R.J., Von Knobelsdorff-Brenkenhoff F., Kramer C.M., Pennell D.J. (2020). Standardized image interpretation and post-processing in cardiovascular magnetic resonance—2020 update. J. Cardiovasc. Magn. Reson..

[17] Bunck A.C., Baeßler B., Ritter C., Kröger J.R., Persigehl T., Santos D.P., Steinmetz M., Niehaus A., Bamberg F., Beer M. (2020). Structured Reporting in Cross-Sectional Imaging of the Heart: Reporting Templates for CMR Imaging of Cardiomyopathies (Myocarditis, Dilated Cardiomyopathy, Hypertrophic Cardiomyopathy, Arrhythmogenic Right Ventricular Cardiomyopathy and Siderosis). RöFo-Fortschritte auf dem Gebiet der Röntgenstrahlen und der bildgebenden Verfahren.

[18] Kawel-Boehm N., Maceira A., Valsangiacomo-Buechel E.R., Vogel-Claussen J., Turkbey E.B., Williams R., Plein S., Tee M., Eng J., A Bluemke D. (2015). Normal values for cardiovascular magnetic resonance in adults and children. J. Cardiovasc. Magn. Reson..

[19] Liu B., Dardeer A.M., Moody W.E., Hayer M.K., Baig S., Price A.M., Leyva F., Edwards N.C., Steeds R.P. (2017). Reference ranges for three-dimensional feature tracking cardiac magnetic resonance: Comparison with two-dimensional methodology and relevance of age and gender. Int. J. Cardiovasc. Imaging.

[20] Cerqueira M.D., Weissman N.J., Dilsizian V., Jacobs A.K., Kaul S., Laskey W.K., Pennell D.J., Rumberger J.A., Ryan T., American Heart Association Writing Group on Myocardial Segmentation and Registration for Cardiac Imaging (2002). Standardized myocardial segmentation and nomenclature for tomographic imaging of the heart. A statement for healthcare professionals from the Cardiac Imaging Committee of the Council on Clinical Cardiology of the American Heart Association. Circulation.

[21] Rosmini S., Bulluck H., Captur G., Treibel T., Abdel-Gadir A., Bhuva A.N., Culotta V., Merghani A., Fontana M., Maestrini V. (2018). Myocardial native T_1_ and extracellular volume with healthy ageing and gender. Eur. Hearth J. Cardiovasc. Imaging.

[22] Sado D.M., Flett A.S., Banypersad S.M., White S.K., Maestrini V., Quarta G., Lachmann R., Murphy E., Mehta A., Hughes D. (2012). Cardiovascular magnetic resonance measurement of myocardial extracellular volume in health and disease. Heart.

[23] Yang E.Y., Ghosn M.G., Khan A., Gramze N.L., Brunner G., Nabi F., Nambi V., Nagueh S.F., Nguyen D.T., Graviss E.A. (2019). Myocardial Extracellular Volume Fraction Adds Prognostic Information Beyond Myocardial Replacement Fibrosis. Circ. Cardiovasc. Imaging.

[24] Delong E.R., Delong D.M., Clarke-Pearson D.L. (1988). Comparing the Areas under Two or More Correlated Receiver Operating Characteristic Curves: A Nonparametric Approach. Biometrics.

[25] Radunski U.K., Lund G.K., Stehning C., Schnackenburg B., Bohnen S., Adam G., Blankenberg S., Muellerleile K. (2014). CMR in patients with severe myocarditis: Diagnostic value of quantitative tissue markers including extracellular volume imaging. J. Am. Coll. Cardiol. Imaging.

[26] Luetkens J.A., Homsi R., Sprinkart A.M., Doerner J., Dabir D., Kuetting D.L., Block W., Andrie R.P., Stehning C., Fimmers R. (2015). Incremental value of quantitative CMR including parametric mapping for the diagnosis of acute myocarditis. Eur. Hearth J. Cardiovasc. Imaging.

[27] Kellman P., Wilson J.R., Xue H., Ugander M., Arai A.E. (2012). Extracellular volume fraction mapping in the myocardium, part 1: Evaluation of an automated method. J. Cardiovasc. Magn. Reson..

[28] Treibel T., Fridman Y., Bering P., Sayeed A., Maanja M., Frojdh F., Niklasson L., Olausson E., Wong T., Kellman P. (2019). Extracellular Volume Associates with Outcomes More Strongly Than Native or Post-Contrast Myocardial T_1_. JACC Cardiovasc. Imaging.

[29] Si-Mohamed S.A., Congi A., Ziegler A., Tomasevic D., Tatard-Leitman V., Broussaud T., Boccalini S., Bensalah M., Rouvière A.-S., Bonnefoy-Cudraz E. (2021). Early Prediction of Cardiac Complications in Acute Myocarditis by Means of Extracellular Volume Quantification with the Use of Dual-Energy Computed Tomography. JACC Cardiovasc. Imaging.

[30] Si-Mohamed S., Restier L., Branchu A., Boccalini S., Congi A., Ziegler A., Tomasevic D., Bochaton T., Boussel L., Douek P. (2021). Diagnostic Performance of Extracellular Volume Quantified by Dual-Layer Dual-Energy CT for Detection of Acute Myocarditis. J. Clin. Med..

[31] Ferreira V.M., Piechnik S.K., Dall’Armellina E., Karamitsos T.D., Francis J.M., Choudhury R.P., Friedrich M.G., Robson M.D., Neubauer S. (2012). Non-contrast T_1_-mapping detects acute myocardial edema with high diagnostic accuracy: A comparison to T_2_-weighted cardiovascular magnetic resonance. J. Cardiovasc. Magn. Reson..

[32] Ferreira V.M., Piechnik S.K., Dall’Armellina E., Karamitsos T.D., Francis J.M., Ntusi N., Holloway C., Choudhury R.P., Kardos A., Robson M.D. (2013). T_1_ Mapping for the diagnosis of acute myocarditis using CMR: Comparison to T_2_-Weighted and late gadolinium enhanced imaging. J. Am. Coll. Cardiol..

[33] Messroghli D.R., Moon J.C., Ferreira V.M., Grosse-Wortmann L., He T., Kellman P., Mascherbauer J., Nezafat R., Salerno M., Schelbert E.B. (2017). Clinical recommendations for cardiovascular magnetic resonance mapping of T_1_, T_2_, T_2_* and extracellular volume: A consensus statement by the Society for Cardiovascular Magnetic Resonance (SCMR) endorsed by the European Association for Cardiovascular Imagi. J. Cardiovasc. Magn. Reson..

[34] Khanna S., Amarasekera A.T., Li C., Bhat A., Chen H.H., Gan G.C., Ugander M., Tan T.C. (2022). The utility of cardiac magnetic resonance imaging in the diagnosis of adult patients with acute myocarditis: A systematic review and meta-analysis. Int. J. Cardiol..

[35] Puntmann V.O., Zeiher A.M., Nagel E. (2018). T_1_ and T_2_ mapping in myocarditis: Seeing beyond the horizon of Lake Louise criteria and histopathology. Expert Rev. Cardiovasc. Ther..

[36] Kotanidis C.P., Bazmpani M.-A., Haidich A.-B., Karvounis C., Antoniades C., Karamitsos T.D. (2018). Diagnostic Accuracy of Cardiovascular Magnetic Resonance in Acute Myocarditis. JACC Cardiovasc. Imaging.

[37] Blissett S., Chocron Y., Kovacina B., Afilalo J. (2019). Diagnostic and prognostic value of cardiac magnetic resonance in acute myocarditis: A systematic review and meta-analysis. Int. J. Cardiovasc. Imaging.

[38] Schumm J., Greulich S., Wagner A., Grün S., Ong P., Bentz K., Klingel K., Kandolf R., Bruder O., Schneider S. (2014). Cardiovascular magnetic resonance risk stratification in patients with clinically suspected myocarditis. J. Cardiovasc. Magn. Reson..

[39] Greulich S., Seitz A., Müller K.A.L., Grün S., Ong P., Ebadi N., Kreisselmeier K.P., Seizer P., Bekeredjian R., Zwadlo C. (2020). Predictors of Mortality in Patients with Biopsy-Proven Viral Myocarditis: 10-Year Outcome Data. J. Am. Hearth Assoc..

[40] Gräni C., Eichhorn C., Bière L., Murthy V.L., Agarwal V., Kaneko K., Cuddy S., Aghayev A., Steigner M., Blankstein R. (2017). Prognostic Value of Cardiac Magnetic Resonance Tissue Characterization in Risk Stratifying Patients with Suspected Myocarditis. J. Am. Coll. Cardiol..

[41] Becker M.A., Cornel J.H., Van de Ven P.M., van Rossum A.C., Allaart C.P., Germans T. (2018). The Prognostic Value of Late Gadolinium-Enhanced Cardiac Magnetic Resonance Imaging in Nonischemic Dilated Cardiomyopathy: A Review and Meta-Analysis. J. Am. Coll. Cardiol..

[42] Polte C.L., Bobbio E., Bollano E., Bergh N., Polte C., Himmelman J., Lagerstrand K.M., Gao S.A. (2022). Cardiovascular Magnetic Resonance in Myocarditis. Diagnostics.

[43] Haaf P., Garg P., Messroghli D.R., Broadbent D.A., Greenwood J.P., Plein S. (2016). Cardiac T_1_ Mapping and Extracellular Volume (ECV) in clinical practice: A comprehensive review. J. Cardiovasc. Magn. Reson..

[44] von Knobelsdorff-Brenkenhoff F., Schüler J., Dogangüzel S., Dieringer M.A., Rudolph A., Greiser A., Kellman P., Schulz-Menger J. (2017). Detection and Monitoring of Acute Myocarditis Applying Quantitative Cardiovascular Magnetic Resonance. Circ. Cardiovasc. Imaging.

[45] Grün S., Schumm J., Greulich S., Wagner A., Schneider S., Bruder O., Kispert E.-M., Hill S., Ong P., Klingel K. (2012). Long-Term Follow-Up of Biopsy-Proven Viral Myocarditis: Predictors of Mortality and Incomplete Recovery. J. Am. Coll. Cardiol..

