# Supplementary material for: Comprehensive Cardiac Magnetic Resonance to Detect Subacute Myocarditis
Source: J Clin Med. 2022 Aug 30;11(17):5113. doi: 10.3390/jcm11175113 (PMC9457022; doi:10.3390/jcm11175113)
Supplement: Supplementary file 1 [file jcm-11-05113-s001.zip › jcm-1832570-supplementary.pdf]

SUPPLEMENTARY FILE S1

CMR image acquisition

All images were acquired in breath hold technique with ECG triggering. Field of View was minimized and adapted to patient's size respectively.

Sequence parameters for functional imaging:

Steady State Free Precession (SSFP) cine loops (repetition time (TR) 3 ms; echo time (TE) 1.5 ms; flip angle 60°, slice thickness 5 mm; matrix 256x192; parallel imaging Generalized Auto-calibrating Partially Parallel Acquisitions (GRAPPA) factor 2; retrospective triggering, 25 frames reconstructed per cardiac cycle) were acquired in standard angulations: four-chamber view (4CV), two-chamber view (2CV) and a stack of short-axis (SAX) slices (gap 5 mm) covering both entire ventricles from base to apex.

Sequence parameters for T_2 mapping:

For myocardial T_2 mapping a product type T_2 prepared SSFP sequence (MyoMaps, SIEMENS Healthcare) with T_2 preparation pulses at 1) none, 2) 24 ms, 3) 55 ms was acquired in basal, mid-ventricular and apical slice. TR 307.5ms, TE 1.17ms, flip angle 20°, slice thickness 8.0mm, baseline matrix 144 x 192.

Sequence parameters for LGE imaging:

2D inversion recovery (IR) gradient recovery echo (GRE) sequence, TR 11 ms, TE 4.4 ms, flip angle 30°, slice thickness 6 mm, baseline matrix 256. The inversion time was adjusted individually to 260-340 ms, to minimize signal from normal myocardium.

Sequence parameters for T_1 and ECV mapping:

A T_1 mapping MOLLI sequence (MyoMaps, SIEMENS Healthcare) with acquisition scheme 5(3)3 (vendor label T_1 long) was acquired native and 15-20min post contrast after Late Gadolinium Enhancement imaging (LGE). Sequence Type Steady State Free Precession (SSFP).

For RR-interval >700ms: TR 280.6ms, TE 1.12ms, flip angle 35°, slice thickness 8.0mm, baseline matrix 169 x 256.

For RR-interval <700ms: TR 360.6 ms, TE 1.12 ms, flip angle 35°, slice thickness 8.0mm, baseline matrix 169 x 256.

Control group

The control group consisted of 15 healthy volunteers with a mean age of 33±5 years (42% female). Controls underwent T_1 and T_2 Mapping in basal, mid-ventricular and apical slice as described. Imaging was performed on the same 1.5 T scanner. Evaluation of Mapping sequences was performed as described in the main document. Global and segmental median of relaxation times and interquartile range was calculated and compared to patients of both the *acute* and the *subacute* group.

Endomyocardial Biopsy Protocol

Endomyocardial biopsies were performed in selected patients according to current ESC diagnostic guidelines [1]. At least five right ventricular biopsies were taken, fixed in 4% phosphate-buffered formaldehyde, and embedded in paraffin. Four µm thick tissue samples were stained with Masson's trichrome, hematoxylin-eosin as well as Giemsa and examined by light microscopy. For immunohistological detection of cardiac immune cells a monoclonal rabbit-anti-CD3 antibody (Clone SP7, 1:500, Novocastra Laboratories, Newcastle upon Tyne, GB), a monoclonal mouse anti-human CD68 antibody (Clone PG-M1, 1:50) and a monoclonal mouse anti-human HLA-DR alpha-chain antibody (clone TAL.1B5, 1:50) both DAKO, Hamburg, Germany was used. Immunohistochemical analysis was performed on an automated immunostainer following the manufacturer's protocol

(Benchmark; Ventana Medical Systems, Tucson, AZ, USA) and using the ultraView detection system (Ventana) and diaminobenzidine as substrate. Tissue sections were counterstained with hematoxylin. The detection of >14 infiltrating leukocytes/mm^2 (including > 7 CD3+ T-lymphocytes and/or CD68+ macrophages) in the presence of myocyte damage and/or fibrosis in addition to enhanced human leukocyte antigen class II expression in professional antigen-presenting immune cells and endothelium was used for the diagnosis of myocarditis [2].

Detection of viral genomes

Deoxyribonucleic acid and ribonucleic acid were extracted with the use of proteinase-K digestion followed by extraction with phenol/chloroform. Nested polymerase chain reaction/reverse transcriptase polymerase chain reaction was performed for the detection of parvovirus B19 (PVB19), Epstein-Barr virus (EBV), and human herpes virus type 6 (HHV6). As control for successful extraction of deoxyribonucleic acid and ribonucleic acid, oligonucleotide sequences were chosen from the glyceraldehyde-3-phosphate-dehydrogenase gene. Specificity of all viral amplification products was confirmed by automatic deoxyribonucleic acid sequencing [2].

References

1. Caforio, A.L.P.; Pankuweit, S.; Arbustini, E.; Basso, C.; Gimeno-Blanes, J.; Felix, S.B.; Fu, M.; Heliö, T.; Heymans, S.; Jahns, R.; et al. Current state of knowledge on aetiology, diagnosis, management, and therapy of myocarditis: A position statement of the European Society of Cardiology Working Group on Myocardial and Pericardial Diseases. *Eur. Heart J.* **2013**, *34*, 2636–2648. https://doi.org/10.1093/eurheartj/eht210.
2. Grün, S.; Schumm, J.; Greulich, S.; Wagner, A.; Schneider, S.; Bruder, O.; Kispert, E.-M.; Hill, S.; Ong, P.; Klingel, K.; et al. Long-Term Follow-Up of Biopsy-Proven Viral Myocarditis: Predictors of Mortality and Incomplete Recovery. *J. Am. Coll. Cardiol.* **2012**, *59*, 1604–1615. https://doi.org/10.1016/j.jacc.2012.01.007.
